# Clinical Outcomes of Volume-Modulated Arc Therapy in 205 Patients with Nasopharyngeal Carcinoma: An Analysis of Survival and Treatment Toxicities

**DOI:** 10.1371/journal.pone.0129679

**Published:** 2015-07-06

**Authors:** Rui Guo, Ling-Long Tang, Yan-Ping Mao, Guan-Qun Zhou, Zhen-Yu Qi, Li-Zhi Liu, Ai-Hua Lin, Meng-Zhong Liu, Jun Ma, Ying Sun

**Affiliations:** 1 Department of Radiation Oncology, Sun Yat-sen University Cancer Center, State Key Laboratory of Oncology in Southern China, Collaborative Innovation Center for Cancer Medicine, Guangzhou, Guangdong, People’s Republic of China; 2 Imaging Diagnosis and Interventional Center, Sun Yat-sen University Cancer Center & State Key Laboratory of Oncology in Southern China, Collaborative Innovation Center for Cancer Medicine, Guangzhou, Guangdong, People’s Republic of China; 3 Department of Medical Statistics and Epidemiology, School of Public Health, Sun Yat-sen University, Guangzhou, Guangdong, People’s Republic of China; Van Andel Institute, UNITED STATES

## Abstract

**Background:**

To investigate the clinical efficacy and treatment toxicity of volume-modulated arc therapy (VMAT) for nasopharyngeal carcinoma (NPC).

**Material and Methods:**

205 VMAT-treated NPC patients from our cancer center were prospectively entrolled. All patients received 68–70 Gy irradiation based on the planning target volume of the primary gross tumor volume. Acute and late toxicities were graded according to the Common Terminology Criteria for Adverse Events v3.0 and Radiation Therapy Oncology Group Late Radiation Morbidity Scoring Criteria.

**Results:**

The median follow-up period was 37.3 months (range, 6.3–45.1 months). The 3-year estimated local failure–free survival, regional failure–free survival, locoregional failure–free survival, distant metastasis–free survival, disease–free survival and overall survival were 95.5%, 97.0%, 94.0%, 92.1%, 86.8% and 97.0%, respectively. Cox regression analysis showed primary gross tumor volume, N stage and EBV-DNA to be independent predictors of VMAT outcomes (P < 0.05). The most common acute and late side effects were grade 2–3 mucositis (78%) and xerostomia (83%, 61%, 34%, and 9% at 3, 6, 12 and 24 months after VMAT), respectively.

**Conclusions:**

VMAT for the primary treatment of NPC achieved very high locoregional control with a favorable toxicity profile. The time-saving benefit of VMAT will enable more patients to receive precision radiotherapy.

## Introduction

Nasopharyngeal carcinoma (NPC) has a distinct epidemiology and geographic distribution, with the highest incidence of 20–50 per 100,000 males in Southeast China [1]. Due to its anatomical features and radiosensitivity, radical radiotherapy has long been the primary treatment for non-disseminated NPC [2]. For decades, the remendous changes of diagnostic and therapeutic techniques have revolutionized the treatment outcomes and quality of life in NPC.

In the management of NPC, intensity-modulated radiation therapy (IMRT) was a breakthrough of radiotherapeutic techniques. The benefits of IMRT in disease control and treatment toxicity have been confirmed in previous reports, due to its dosimetric advantages [3–5]. Nonetheless, several issues must be addressed with the widespread adoption of conventional IMRT (c-IMRT). First of all, the number of patients who can received precision radiotherapy may be limited by the prolonged treatment time. Second, the treatment accuracy of c-IMRT may be worsen due to increased intra-fractional patient motion caused by the prolonged delivery time [6]. Furthermore, the possibly risk of radiation-induced secondary cancers may be increased as a result of the raised peripheral dose and monitor units of c-IMRT [7–9].

Volumetric-modulated arc therapy (VMAT) was proposed to overcome the drawbacks of c-IMRT. With VMAT, the multi-leaf collimator leaf positions, dose rate and gantry rotation speed can be continuously modulated during a single 360° rotation [10]. Planning studies have reported that VMAT-based treatment plans are similar or better than c-IMRT-based plans, with fewer monitor units and shorter delivery times for head-and-neck cancers [11–13]. Our previous study also investigated the differences of dosimetric distribution, delivery time and low dose burdens between VMAT and c-IMRT [14]. However, the clinical benefits of short treatment time are unclear. Furthermore, the treatment toxicity of VMAT are not known for NPC which is distinct from other head and neck cancers in its epidemiology, pathology and clinical attributes.

Therefore, we conducted this research to investigate suvival and treatment toxicity of VMAT in NPC patients. Furthmore, we revaluated the prognostic factors for the failure model of NPC patients treated with VMAT.

## Materials and Methods

### Patient Characteristics

From August 2010 to June 2011, 205 consecutive biopsy-proven, non-metastatic NPC patients who underwent VMAT at Sun-Yat Sen Cancer Center were enrolled in this were enrolled in this prospective observational study. This study was approved by the the ethics committee of Sun-Yat Sen Cancer Center, and the participants provided written consent before undergoing radiotherapy. Data from physical examinations, imaging and therapeutic schedules were obtained for all patients, along with information on any acute and late normal-tissue effects. The clinical characteristics are listed in Table 1.

**Table 1:** Clinical features of the 205 NPC patients in this study.

Patient	characteristics	No. (%)
**Age**		
	Median	45 years
	Range	21–75 years
**Gender**		
	Male	157 (76.6)
	Female	48 (23.4)
**Histology**		
	WHO type II	1 (0.5)
	WHO type III	204 (99.5)
**T classification** *		
	T1	31 (15.1)
	T2	32(15.6)
	T3	91 (44.4)
	T4	51 (24.9)
**N classification** *		
	N0	44 (21.5)
	N1	105 (51.2)
	N2	40 (19.5)
	N3	16(7.8)
**Clinical Stages** *		
	I	13 (6.3)
	II	35 (17.1)
	III	94 (45.9)
	IV	63 (30.7)
**Therapy**		
	Chemotherapy in stage III- IV	141 (89.8)
	Boost in stage T3–T4 patients	2 (1.4)

Abbreviations: NPC = nasopharyngeal carcinoma;VMAT = volume-modulated arc therapy; c-IMRT = conventional intensity modulated radiation therapy

* According to the 7th AJCC/UICC staging system

Pretreatment evaluations consisted of a complete physical examination, hematologic and biochemistry profiles, nasopharyngeal fiberoptic endoscopy, magnetic resonance imaging (MRI) and of the nasopharyngeal and cervical regions, chest radiography, bone scintigraphy, abdominal ultrasonography and positron emission tomography. All patients were restaged according to the 7th UICC/AJCC staging system (Table 1).

### Simulation, immobilization and Delineation

All patients were immobilized in a supine position, using a thermoplastic mask covering the head, neck and shoulder. CT was performed after intravenous contrast administration, and 3-mm slices were obtained from the head to 2 cm below the sternoclavicular joint.

Target volumes were defined in accordance with the International Commission on Radiation Units and Measurements reports 50 and 62 as described previously [14]. Gross tumor volume (GTV) was defined as the tumor volume determined from MRI, clinical records and endoscopic findings, including GTVnx and GTVnd. GTVnx included the sum of the primary tumor volume and the enlarged retropharyngeal nodes, while GTVnd was the volume of clinically involved gross lymph nodes. Clinical tumor volumes (CTVs) were individually delineated based on the tumor-invasion pattern [15]. CTV1 and CTV2 represented high- and low-risk regions for microscopic extension, respectively.

### Radiotherpay

A standard constraint set comprising Radiation Therapy Oncology Group (RTOG) 0615 and RTOG 0225 was used for optimization and evaluation (Supplementary information)[14]. A planning target volume (PTV) was created by adding a three-dimensional margin of 3 mm to the delineated target volume to compensate for the variabilities in treatment set-up and internal organ motion. For VMAT, dose optimization and calculation were performed on the Monaco treatment planning system (version 3.02; Elekta Medical Systems, Crawley, UK) using the Monte Carlo algorithm. VMAT plans were generated for a 6-MV Elekta Synergy linear accelerator equipped with the Elekta Precise Beam VMAT linac control system and a conventional 80 leaf MLC (1 cm leaf width at iso-center). All plans were generated by a team of dosimetrists by using a whole-field (including neck-radiation) simultaneous integrated-boost technique. All the VMAT plans used a single complementary coplanar arc of 360° with the couch angle set to 0°.

The dose was set at 68–70, 64–70, 60–62 and 54–56 Gy, in 30–33 fractions, for the PTVs derived from GTVnx, GTVnd, CTV1 and CTV2, respectively. Radiation was delivered once daily, at 5 fractions per week. All targets were treated simultaneously using the simultaneous integrated boost technique. A boost portal was used in the event of persistent disease. Three patients had residual tumors 3 months after treatment, and underwent intracavitary brachytherapy with iridium-192 (15–20 Gy, 3–5 fractions, delivered in 2 weeks).

### Chemotherapy

Overall, 89.8% (141/157) patients with stage III or IVa-b disease (T3–T4 or N2–N3) received chemotherapy, including concomitant chemoradiotherapy with or without inductive/adjuvant chemotherapy. Reasons for deviation from the guidelines included age, organ dysfunction and allergic reactions that indicated intolerance to chemotherapy. Inductive or adjuvant chemotherapy consisted of cisplatin with 5-fluorouracil, cisplatin with taxoids or a triplet of cisplatin, 5-fluorouracil and taxoids every 3 weeks for two-to-three cycles. Concomitant chemotherapy consisted of cisplatin given in weeks 1, 4 and 7 of radiotherapy or cisplatin given weekly.

### Patient assessment and follow-up

During radiotherapy, all patients underwent standardized follow-up with weekly evaluations by a radiation oncologist and complete physical examination, hematologic and biochemistry profiles every week and fiberoptic endoscope examination every 2 weeks. Treatment responses were evaluated according to the Response Evaluation Criteria in Solid Tumors guidelines (version 1.1)[16]. The Common Terminology Criteria for Adverse Events version 3.0 was used for grading acute toxicity, and the RTOG Late Radiation Morbidity Scoring Criteria were used for grading late toxicity.

Post-treatment assessment included nasopharyngeal endoscopy, MRI, chest radiography, abdominal ultrasonography and bone scintigraphy approximately every 3 months in the first 2 years, every 6 months in the next 3 years and annually thereafter. A follow-up MRI of the nasopharynx and neck was performed to document therapeutic response and determine whether the patient was clinically disease free or required further diagnostic biopsy and/or treatment.

### Statistical methods

The probabilities of disease-free survival (DFS), overall survival (OS), distant metastasis–free survival (DMFS), local relapse–free survival (LRFS), regional relapse–free survival (RRFS) and locoregional relapse–free survival (LRRFS) were estimated using the Kaplan–Meier method. The follow-up duration was calculated from the first day of therapy to the day of death or last examination. Multivariate analysis was performed using Cox’s proportional hazard model for the entire cohort, to examine the impact of various prognostic factors, including host factors (age, sex, WHO histological grade), tumor factors (T and N stage), primary GTVnx (GTV-P), Epstein–Barr virus (EBV)-DNA and treatment factors (chemotherapy).

## Results

### Dose–volume analysis for target coverage and organs at risk

The dose–volume histograms for the target volumes and organs at risk (OARs) are shown in Tables 2 and 3. On average, the target volumes had excellent coverage: 99.73% of the PTV-GTV, 99.83% of the PTV-CTV1 and 99.04% of the PTV-CTV2 received ≥95% of the prescribed dose. The average dose to 1% of the planning OAR volume of the spinal cord and brainstem was 44.68 and 56.35 Gy, respectively. The doses delivered were within the tolerance limits of critical normal structures, except the parotid gland (Table 3).

**Table 2:** Mean (± SD) of doses based on the planning tumor volume for the 205 patients treated with volume-modulated arc therapy Index.

	PGTVnx	PCTV1	PCTV2
**D1 (Gy)** ^**§**^	76.72±1.89	75.97±2.50	74.95±1.83
**D99 (Gy)** ^**#**^	67.86±1.91	60.63±2.38	51.81±2.62
**D95** ^**‡**^	69.82±1.45	63.34±2.00	55.69±2.01
**Dmean(Gy)**	72.97±1.37	69.66±2.06	62.84±2.14
**V93** ^**¶**^	99.89±0.38	99.93±0.18	99.37±0.51
**V95** ^**£**^	99.73±0.70	99.83±0.45	99.04±0.75
**V100** *	97.79±1.29	99.05±1.05	97.17±2.02
**V110** ^**†**^	5.65±6.09	—	—
**V115** ^**&**^	0.11±0.43	—	—

Abbreviations: PGTVnx = planning target volume for gross tumor volume; PCTV1 = planning target volume for high-risk clinical tumor volumes; PCTV2 = planning target volume for low-risk clinical tumor volumes

§: Dose received by 1% of the volume

#: Dose received by 99% of the volume

¶: Percentage volume covering 93% of the Rx (prescribed dose)

£: Percentage volume covering 95% of the Rx (prescribed dose)

*: Percentage volume covering 100% of the Rx (prescribed dose)

†: Percentage volume that received >110% of the Rx (prescribed dose)

&: Percentage volume that received >115% of the Rx (prescribed dose)

‡: Dose received by 95% of the volume

**Table 3:** Mean (± SD) of the doses for organs at risk for the 205 patients treated with volume-modulated arc therapy.

Organ	Dose index	Dose
**Spinalcord**	Dmax(Gy)	44.43±2.29
**Spinalcord PRV**	D1(Gy) ^¶^	44.68±1.91
**Brain stem**	Dmax(Gy)	56.81±5.52
**Brain stem PRV**	D1(Gy) ^¶^	56.35±4.87
**Optic nerves-L**	Dmax(Gy)	41.89±17.05
**Optic nerves-L PRV**	D1(Gy) ^¶^	41.02±16.76
**Optic nerves-R**	Dmax(Gy)	41.25±17.33
**Optic nerves-R PRV**	D1(Gy) ^¶^	40.71±17.51
**Optic chiasm**	Dmax(Gy)	47.65±15.70
**Optic chiasm PRV**	D1(Gy) ^¶^	47.53±14.43
**Lens-L**	Dmax(Gy)	7.24±3.04
**Lens-R**	Dmax(Gy)	7.29±3.10
**Parotid gland-L**	Dmean	38.89±6.10
	V30(%)^§^	65.42±18.08
	V20 (cc) *	2.44±3.82
**Parotid gland-R**	Dmean	39.84±6.59
	V30(%)^§^	67.85±17.68
	V20(cc) *	2.50±4.68
**Temporal lobe-L**	D1^¶^	59.48±7.96
**Temporal lobe-R**	D1^¶^	59.76±8.47
**Mandible-L**	D1cc^&^	55.65±7.67
**Mandible-R**	D1cc^&^	56.04±5.95
**TM joint-L**	D1cc^&^	41.79±10.03
**TM joint-R**	D1cc^&^	42.50±9.29
**Larynx**	Dmean	48.05±5.17
**Inner ears-L**	Dmean	43.33±8.76
**Inner ears-R**	Dmean	42.57±8.14

Abbreviations: L-left; R-right PRV: planing risk volume

¶: Dose received by 1% of the volume.

§: Percentage volume of at least one gland which received >30 Gy radiation.

*: Volume of both glands which received <20 Gy radiation.

&: Dose received by 1cm^3^ of the volume.

### Clinical outcomes

The median follow-up period was 37.3 months (range, 6.3–45.1 months). The 3-year estimated LRFS, RRFS, LRRFS, DMFS, DFS and OS rates were 95.5%, 97.0%, 94.0%, 92.1%, 86.8% and 97.0%, respectively. Local, regional and distant failure occurred in 9, 6 and 16 patients, respectively. Of the nine local failures, eight occurred within the 95% isodose lines and were considered in-field failures. The remaining failure was a marginal miss. All six regional failures occurred well within the 95% isodose lines and were in-field failures. Among the 16 patients with distant metastases, 10 developed metastasis in a single organ, and 6 had multi-organ metastases. There were ten, six, four and three cases of bone, liver, lung and distant lymph node metastases, respectively.

### Prognostic factors

The value of various potential prognostic factors on for predicting DFS, OS, DMFS and LRRFS was evaluated. Univariate analysis showed significant associations of that N classificationstage, GTV-P and EBV—DNA and were significantly associated with DFS (P<0.05), N stage and GTV-P with DMFS and GTV-P were significantly associated with OS and LRRFS (P<0.05), N classification, GTV-P were significantly associated with DMFS (P<0.05). GTV-P were significantly associated with LRRFS (P<0.05) (Table 4).

**Table 4:** Univariate analysis for various clinical endpoints.

Characteristic	N	DFS*	*P*	OS*	*P*	DMFS*	*P*	LRRFS*	*P*
**Age**			0.106		0.011		0.057		0.913
** ≤45 year**	108	83.3		94.4		88.7		94.3	
** >45 year**	97	90.7		100		95.9		93.8	
**Sex**			0.163		0.112		0.484		0.158
** male**	153	85.0		96.0		91.4		92.6	
** female**	52	92.3		100.0		94.2		98.1	
**Histology**			0.801		0.602		0.202		0.308
** WHO type II**	18	88.9		100		100		88.9	
** WHO type III**	187	86.6		96.8		91.3		94.5	
**Family history**			0.389		0.269		0.845		0.062
** without**	137	85.4		96.3		91.9		91.8	
** with**	68	89.7		98.5		92.6		98.5	
**T classification**			0.314		0.445		0.733		0.209
** T1**	31	96.8		100		96.8		100	
** T2**	32	81.3		96.7		90.6		87.5	
** T3**	91	85.7		95.6		92.2		94.2	
** T4**	51	86.3		98.0		90.2		94.1	
**N classification**			0.001		0.858		<0.001		0.549
** N0**	44	95.5		100		97.7		95.5	
** N1**	105	90.5		97.1		97.1		95.1	
** N2**	40	77.5		97.5		82.4		92.4	
** N3**	16	62.5		93.3		68.8		87.1	
**GTV volume**			0.002		0.008		0.051		0.011
** ≤19 cc**	122	92.6		99.2		95.0		97.5	
** >19 cc**	83	78.3		93.9		87.7		88.7	
**EBVDNA**			<0.001		0.077		<0.001		0.077
** ≤5.0×10** ^**3**^ **copy**	146	93.8		98.6		97.2		95.9	
** >5.0×10** ^**3**^ **copy**	59	69.5		93.1		79.3		89.4	

Abbreviations: N: number; p: p value; DFS: disease-free survival; OS: overall survival; DMFS: distant metastasis-free survival; LRRFS: local regional relapse-free survival

*According to survival rates of three years

Multivariate analysis revealed the following independent prognostic predictors (P < 0.05): GTV-P (hazard ratio [HR]: 3.018), N stage (HR: 3.352) and EBV-DNA (HR: 3.422) for DFS; GTV-P (HR: 2.981), N stage (HR: 7.028) and EBV-DNA (HR: 5.038) for DMFS; and GTV (HR: 3.962) for LRRFS (Table 5).

**Table 5:** Multivariate analysis of the impact of all variables on survival.

Endpoint	Variable	HR	95% CI	P-value‡
**Disease-free survival**	GTV	3.018	1.328–6.859	0.008
	N classification*	3.352	1.491–7.539	0.003
	EBVDNA	3.422	1.462–8.008	0.005
	Age	0.455	0.203–1.022	0.057
**Overall survival**	GTV	10.038	0.979–102.892	0.052
**Distant metastasis-free survival**	GTV	2.981	1.063–8.356	0.038
	N classification*	7.028	2.206–22.392	0.001
	EBVDNA	5.038	1.599–15.871	0.006
	Age	0.282	0.089–0.889	0.031
**Local regional relapse-free survival**	GTV	3.962	1.065–14.741	0.040

Abbreviations: GTV: gross tumor volume; CI, confidence interval; HR, hazards ratio; EBV: Plasma Epstein-Barr virus

*According to the 7th AJCC/UICC staging system.

‡ Multivariate *P* values were calculated using an adjusted Cox proportional-hazards model. The following parameters were included in the Cox proportion hazard model by backward elimination: age (≤45 vs. >45 year), gender (male vs.female), WHO histological grade, T classification (T1-2 vs. T3-4), N classification (N0-1 vs. N2-3), use of chemotherapy (with vs. without), GTV (≤19 cc vs. >19cc), EBVDNA (≤5.0×10^3^ copy vs. >5.0×10^3^ copy).

### Acute and late toxicities

In all VMAT patients, acute side effects were well tolerated (Table 6). The most common acute side effects were dermatitis and mucositis, with RTOG grade 2 or 3 dermatitis and mucositis occuring in 34% and 78% patients, respectively. The most common late side effect was xerostomia, whose incidence decreased with time. We found that 83%, 61%, 34% and 9% patients experienced RTOG grade 2 or 3 xerostomia at 3, 6, 12 and 24 months after VMAT, respectively. Six (3%) patients developed RTOG grade 3 hearing loss, and seven (3.5%) had MRI-diagnosed temporal lobe necrosis (TLN). All these patients with TLN had stage T3–4 disease, with wide skull base erosion or intracranial invasion (Table 6).

**Table 6:** Frequency of worst acute toxicity and late toxicity in 205 nasopharyngeal carcinoma patients treated with volume-modulated arc therapy.

Grade	Grade 0	Grade 1	Grade 2	Grade 3	Grade 4
**Acute Toxicity** *	Cases (%)	Cases (%)	Cases (%)	Cases (%)	Cases (%)
Dermatitis	—	135 (66)	60 (29)	10 (5)	0 (0)
Mucositis	—	45 (22)	103 (50)	57 (28)	0 (0)
Dry mouth	—	55 (27)	121 (59)	21 (10)	0 (0)
Vomiting	—	57 (28)	25 (12)	21 (10)	0 (0)
Leucopenia	—	33 (16)	16 (8)	8 (4)	0 (0)
Neutropenia	—	22 (12)	22 (12)	6 (3)	0 (0)
Anaemia	—	49(24)	12 (6)	4 (2)	0 (0)
Thrombocytopenia	—	16 (8)	4 (2)	4 (2)	0 (0)
Fever	—	8 (4)	4 (2)	2 (1)	0 (0)
Weight loss	—	71 (35)	6 (3)	0 (0)	0 (0)
Liver function derangement	—	27 (13)	4 (2)	0 (0)	0 (0)
**Late toxicity** ^**&**^					
Xerostomia	53(26)	133(65)	14(7)	4(2)	0(0)
Ear	111(54)	58(28)	30(15)	6(3)	0(0)
Cranial nerve	202(98.5)	1(0.5)	1(0.5)	1(0.5)	0(0)
Spinal cord	198(96)	6(3)	1(0.5)	0(0)	0(0)
Mandible	197(96)	6(3)	2(1)	0(0)	0(0)
Skin	170(83)	31(15)	4(2)	0(0)	0(0)
Subcutaneous tissue	174(85)	27(13)	4(2)	0(0)	0(0)

* graded according to the Common Terminology Criteria for Adverse Events v3.0 (CTCAE 3.0)

& graded according to Radiation Therapy Oncology Group (RTOG) Late Radiation Morbidity Scoring Criterion

## Discussion

To our knowledge, this is the first clinical study on VMAT in a large cohort of NPC patients. Unlike other head-and-neck malignancies, NPC requires greater sparing of critical normal structures and encompasses the entire neck lymph nodal chain down to the supraclavicular fossa. Thus, treatment planning for NPC is challenging, and we believe that our findings will be useful for future studies on and treatment strategies for NPC.

With our VMAT protocol, optimal target coverage was achieved. Recent studies have shown that VMAT can achieve V95 values (volume receiving 95% of prescribed dose) of 96%–98.9% in the PTV for GTV [11–13]. We achieved a V95 close to 100% (99.73%), which is excellent and consistent with previous studies. Among critical normal structures, the parotid gland and larynx did not meet dose constraints, which is similar to the study by Johnston et al and our previous study [17, 18]. This is attributable to the partial overlap of deep lobes of parotid gland with the PTV and the need for upper cervical nodal coverage with high-to-intermediate dose volumes. Our previous study have showed that the average treatment time for VMAT was shorter than c-IMRT (424s ± 64 s vs. 778 ± 126 s; P < 0.05) [14].

Our reported LRFS (95.5%), RRFS (97.0%), DMFS (93.1%) and OS (97.0%) are excellent and similar to those of c-IMRT [3, 5, 19–22] (Table 7). Case series from early adopters of IMRT have shown it to be safe and effective for NPC treatment. Wolden et al. reported that the 3-year actuarial rates of LRFS, RFRS, DMFS and OS were 91%, 93%, 78% and 83%, respectively, in 74 NPC patients [22]. Wong et al. reported that the 3-year actuarial rates of LRFS, RRFS, DMFS and OS were 94%, 93%, 87% and 87%, respectively, in 175 NPC patients [20]. Overall, the reported 3-year LRFS, RRFS, DMFS and OS were 90%–95%, 91%–98%, 78%–91% and 87%–94%, respectively. However, the OS at our center was 98.0%, which is higher than that reported by others. We think that this is attributable to (i) advances in concurrent chemoradiotherapy for recurrent and distant NPC; (ii) timely and effective supportive treatment during radiotherapy, which may have reduced treatment-related deaths; and (iii) timely and efficient follow-up, which enabled the early detection and treatment of small lesions.

**Table 7:** Treatment parameters and outcomes of intensity-modulated radiotherapy.

Author	N	Technique	T3-4	Total	Dose/Fraction	Time	LFS	NFS	DMFS	OS
			(%)	Dose(Gy)	(Gy)	(year)	(%)	(%)	(%)	(%)
**Bakst**	25	IMRT	28	70.2	2.34	3	91	91	91	89
**Kam[5]**	63	IMRT	51	66	2	3	92	98	79	90
**Wolden[22]**	74	IMRT	51	70.2	2.34	3	91	93	78	83
**Lin[21]**	326	IMRT	61	66–69.8	2.2–2.25	3	95	98	90	90
**Tham[3]**	195	IMRT	NS	70	2.12	3	90	—	89	94
**Wong[20]**	175	IMRT	35	66–70	2–2.12	3	94	93	87	87
**Current study**	205	VMAT	67	68–70	2.12–2.24	2	95.5	97	93.1	98

Abbreviations: N: number, LFS: local failure-free survival, RFS: regional failure-free survival, DMFS: distant metastasis-free survival, OS: overall survival

We evaluated the value of various potential prognostic factors, including EBV-DNA, GTV-P, age and sex, for predicting DFS, OS, DMFS and LRRFS. All IMRT series thus far have reported that the current T classification of the TNM staging system is not useful for segregating patients into at-risk groups [5, 23]. This was confirmed in our study. GTV-P, which reflects tumor bulk, was associated with advanced disease, poor prognosis, distant metastasis and local recurrence in IMRT-treated NPC patients. We used the cut-off point of the GTV-P (≥19 ml vs. <19 ml), according to our previous study [24].

EBV-DNA was also confirmed using predefined cutoffs as an independent prognostic marker of NPC, which is consistent with previous research [25]. We defined the ideal cutoff as 5000 copies, by maximizing the conditional Youden score by using a receiver operating characteristic curve. In VMAT-treated patients, higher EBV-DNA copies adversely affected DMFS and DFS. Additionally, the N stage was an independent predictor of DMFS and DFS.

All our patients tolerated VMAT well. The most common acute toxicity was grade 2–3 mucositis (78% patients). This is excellent compared to 91%–93% toxicity rates in other reports [4, 5]. The most common late toxicity was xerostomia, which was caused by high-dose radiation to the major salivary glands. c-IMRT can reduce the dose to the salivary glands, while simultaneously delivering a high dose to the tumor [26]. Wolden et al. detected long-term grade 2–3 xerostomia in 32% of 59 NPC patients after 12 months of follow-up [22]. In our study, 34% patients experienced grade 2–3 xerostomia at 12 months after VMAT. The rates of MRI-diagnosed TLN vary from 4.6% to 7.5% [27–29]; the median latency for TLN detection is 30 months (range, 6–56 months) [29]. In our study, only 3.5% patients had MRI-diagnosed TLN, due to the short follow-up. Our results showed that TLN is most likely in patients with advanced T-stage. One possible reason for this is that in patients with wide skull base erosion or intracranial invasion, neurological organs are likely to be partially included in the radiation fields to achieve satisfactory target coverage and unavoidably receive high-dose irradiation.

The mean follow-up time in our study was short. Although we reported 3-year survival results, we need to closely follow up the patients and report the 5-year results to fully assess survival and late toxicity. Long-term benefits on clinical outcomes are expected because of the short treatment time.

## Conclusions

VMAT for the primary treatment of NPC achieved very high locoregional control with a favorable toxicity profile at early follow-up, which is similar to c-IMRT outcomes. With the time-saving benefit of VMAT, more patients can undergo precision radiotherapy. Our findings are useful for future studies on and treatment strategies for NPC.

## Supporting Information

S1 Table Standard constraint set used for optimization and evaluation. (DOC)

## References

[1] Jemal A, Bray F, Center MM, Ferlay J, Ward E, Forman D. Global cancer statistics. CA Cancer J Clin. 2011;61(2):69–90. 10.3322/caac.20107 . 21296855

[2] Wei WI, Sham JS. Nasopharyngeal carcinoma. Lancet. 2005;365(9476):2041–54. 10.1016/S0140-6736(05)66698-6 . 15950718

[3] Tham IW, Hee SW, Yeo RM, Salleh PB, Lee J, Tan TW, et al Treatment of nasopharyngeal carcinoma using intensity-modulated radiotherapy-the national cancer centre singapore experience. International journal of radiation oncology, biology, physics. 2009;75(5):1481–6. 10.1016/j.ijrobp.2009.01.018 . 19386431

[4] Lee N, Xia P, Quivey JM, Sultanem K, Poon I, Akazawa C, et al Intensity-modulated radiotherapy in the treatment of nasopharyngeal carcinoma: an update of the UCSF experience. International journal of radiation oncology, biology, physics. 2002;53(1): 12–22. doi: S0360301602027244 [pii]. . 12007936 10.1016/s0360-3016(02)02724-4

[5] Kam MK, Teo PM, Chau RM, Cheung KY, Choi PH, Kwan WH, et al Treatment of nasopharyngeal carcinoma with intensity-modulated radiotherapy: the Hong Kong experience. International journal of radiation oncology, biology, physics. 2004;60(5): 1440–50. 10.1016/j.ijrobp.2004.05.022 . 15590175

[6] Hoogeman MS, Nuyttens JJ, Levendag PC, Heijmen BJ. Time dependence of intrafraction patient motion assessed by repeat stereoscopic imaging. International journal of radiation oncology, biology, physics. 2008;70(2):609–18. doi: S0360-3016(07) 04149–1 [pii] 10.1016/j.ijrobp.2007.08.066 . 17996389

[7] Hall EJ. Intensity-modulated radiation therapy, protons, and the risk of second cancers. International journal of radiation oncology, biology, physics. 2006;65(1):1–7. 10.101/j.ijrobp.2006.01.027 . 16618572

[8] Hall EJ, Wuu CS. Radiation-induced second cancers: the impact of 3D-CRT and IMRT. International journal of radiation oncology, biology, physics. 2003;56(1):83–8. doi: S0360301603000737 [pii]. . 12694826 10.1016/s0360-3016(03)00073-7

[9] Patil VM, Kapoor R, Chakraborty S, Ghoshal S, Oinam AS, Sharma SC. Dosimetric risk estimates of radiation-induced malignancies after intensity modulated radiotherapy. J Cancer Res Ther. 2010;6(4):442–7. 10.4103/0973-1482.77082 . 21358077

[10] Yu CX. Intensity-modulated arc therapy with dynamic multileaf collimation: an alternative to tomotherapy. Phys Med Biol. 1995;40(9):1435–49. . 8532757 10.1088/0031-9155/40/9/004

[11] Verbakel WF, Cuijpers JP, Hoffmans D, Bieker M, Slotman BJ, Senan S. Volumetric intensity-modulated arc therapy vs. conventional IMRT in head-and-neck cancer: a comparative planning and dosimetric study. International journal of radiation oncology, biology, physics. 2009;74(1):252–9. 10.1016/j.ijrobp.2008.12.033 . 19362244

[12] Bertelsen A, Hansen CR, Johansen J, Brink C. Single Arc Volumetric Modulated Arc Therapy of head and neck cancer. Radiotherapy and oncology: journal of the European Society for Therapeutic Radiology and Oncology. 2010;95(2):142–8. doi: S0167-8140 (10)00062-9 [pii] 10.1016/j.radonc.2010.01.011 . 20188427

[13] Doornaert P, Verbakel WF, Bieker M, Slotman BJ, Senan S. RapidArc planning and delivery in patients with locally advanced head-and-neck cancer undergoing chemoradiotherapy. International journal of radiation oncology, biology, physics. 2011;79(2):429–35. 10.1016/j.ijrobp.2009.11.014 . 20421159

[14] Sun Y, Guo R, Yin WJ, Tang LL, Yu XL, Chen M, et al Which T category of nasopharyngeal carcinoma may benefit most from volumetric modulated arc therapy compared with step and shoot intensity modulated radiation therapy. PloS one. 2013;8(9):e75304 10.1371/journal.pone.0075304 . 24086503 PMC3783380

[15] Liang SB, Sun Y, Liu LZ, Chen Y, Chen L, Mao YP, et al Extension of local disease in nasopharyngeal carcinoma detected by magnetic resonance imaging: improvement of clinical target volume delineation. International journal of radiation oncology, biology, physics. 2009; 75(3): 742–50. 10.1016/j.ijrobp.2008.11.053 . 19251378

[16] Eisenhauer EA, Therasse P, Bogaerts J, Schwartz LH, Sargent D, Ford R, et al New response evaluation criteria in solid tumours: revised RECIST guideline (version 1.1). Eur J Cancer. 2009;45(2):228–47. 10.1016/j.ejca.2008.10.026 . 19097774

[17] JJohnston M, Clifford S, Bromley R, Back M, Oliver L, Eade T. Volumetric-modulated arc therapy in head and neck radiotherapy: a planning comparison using simultaneous integrated boost for nasopharynx and oropharynx carcinoma. Clin Oncol (R Coll Radiol). 2011;23(8):503–11. 10.1016/j.clon.2011.02.002 . 21397477

[18] Dijkema T, Raaijmakers CP, Ten Haken RK, Roesink JM, Braam PM, Houweling AC, et al Parotid gland function after radiotherapy: the combined michigan and utrecht experience. International journal of radiation oncology, biology, physics. 2010;78 (2): 449–53. 10.1016/j.ijrobp.2009.07.1708 . 20056347 PMC2889151

[19] Bakst RL, Lee N, Pfister DG, Zelefsky MJ, Hunt MA, Kraus DH, et al Hypofractionated dose-painting intensity modulated radiation therapy with chemotherapy for nasopharyngeal carcinoma: a prospective trial. International journal of radiation oncology, biology, physics. 2011;80(1):148–53. 10.1016/j.ijrobp.2010.01.026 . 20605352 PMC2952060

[20] Wong FC, Ng AW, Lee VH, Lui CM, Yuen KK, Sze WK, et al Whole-field simultaneous integrated-boost intensity-modulated radiotherapy for patients with nasopharyngeal carcinoma. International journal of radiation oncology, biology, physics. 2010;76(1):138–45. 10.1016/j.ijrobp.2009.01.084 . 19646824

[21] Lin S, Pan J, Han L, Zhang X, Liao X, Lu JJ. Nasopharyngeal carcinoma treated with reduced-volume intensity-modulated radiation therapy: report on the 3-year outcome of a prospective series. International journal of radiation oncology, biology, physics. 2009;75(4):1071–8. 10.1016/j.ijrobp.2008.12.015 . 19362784

[22] Wolden SL, Chen WC, Pfister DG, Kraus DH, Berry SL, Zelefsky MJ. Intensity-modulated radiation therapy (IMRT) for nasopharynx cancer: update of the Memorial Sloan-Kettering experience. International journal of radiation oncology, biology, physics. 2006;64(1):57–62. 10.1016/j.ijrobp.2005.03.057 . 15936155

[23] Ng WT, Lee MC, Hung WM, Choi CW, Lee KC, Chan OS, et al Clinical outcomes and patterns of failure after intensity-modulated radiotherapy for nasopharyngeal carcinoma. International journal of radiation oncology, biology, physics. 2011;79(2):420–8. 10.1016/j.ijrobp.2009.11.024 . 20452132

[24] Guo R, Sun Y, Yu XL, Yin WJ, Li WF, Chen YY, et al Is primary tumor volume still a prognostic factor in intensity modulated radiation therapy for nasopharyngeal carcinoma? Radiotherapy and oncology: journal of the European Society for Therapeutic Radiology and Oncology. 2012;104(3):294–9. 10.1016/j.radonc.2012.09.001 . 22998947

[25] Wang WY, Twu CW, Chen HH, Jiang RS, Wu CT, Liang KL, et al Long-term survival analysis of nasopharyngeal carcinoma by plasma Epstein-Barr virus DNA levels. Cancer. 2013;119(5):963–70. 10.1002/cncr.27853 . 23065693

[26] Pow EH, Kwong DL, McMillan AS, Wong MC, Sham JS, Leung LH, et al Xerostomia and quality of life after intensity-modulated radiotherapy vs. conventional radiotherapy for early-stage nasopharyngeal carcinoma: initial report on a randomized controlled clinical trial. International journal of radiation oncology, biology, physics. 2006;66(4):981–91. 10.1016/j.ijrobp.2006.06.013 . 17145528

[27] Zhou GQ, Yu XL, Chen M, Guo R, Lei Y, Sun Y, et al Radiation-induced temporal lobe injury for nasopharyngeal carcinoma: a comparison of intensity-modulated radiotherapy and conventional two-dimensional radiotherapy. PloS one. 2013;8(7):e67488 10.1371/journal.pone.0067488 . 23874422 PMC3707870

[28] Zeng L, Tian YM, Sun XM, Chen CY, Han F, Xiao WW, et al Late toxicities after intensity-modulated radiotherapy for nasopharyngeal carcinoma: patient and treatment-related risk factors. British journal of cancer. 2014;110(1):49–54. 10.1038/bjc.2013.720 . 24253503 PMC3887308

[29] Su SF, Han F, Zhao C, Chen CY, Xiao WW, Li JX, et al Long-term outcomes of early-stage nasopharyngeal carcinoma patients treated with intensity-modulated radiotherapy alone. International journal of radiation oncology, biology, physics. 2012;82(1):327–33. 10.1016/j.ijrobp.2010.09.011 . 21035959

