# Supplementary material for: Clinical Outcomes of Volume-Modulated Arc Therapy in 205 Patients with Nasopharyngeal Carcinoma: An Analysis of Survival and Treatment Toxicities
Source: PLoS One. 2015 Jul 6;10(7):e0129679. doi: 10.1371/journal.pone.0129679 (PMC4492500; doi:10.1371/journal.pone.0129679)
Supplement: S1 Table — (DOC) [file pone.0129679.s001.doc]

**S1 table: Standard constraint set used for optimization and evaluation**

Abbreviations: PTV, planning target volume; PRV, planning risk volume; L, light; R, right;

| **Structure** | **Objective** | |
| --- | --- | --- |
| **PTV_7000** | V93%^¶^ | ≥99% |
|  | V100%^#^ | ≥95% |
|  | V110%^†^ | ≤20% |
|  | V115% || | ≤5% |
| **PTV_6000** | V93%^¶^ | ≥99% |
|  | V100%^#^ | ≥95% |
| **PTV_5600** | V93%^¶^ | ≥99% |
|  | V100%^#^ | ≥95% |
| **SpinalCord** | Max | ≤45Gy |
| **SpinalCord_PRV** | D1^‡^ | ≤50 Gy |
| **BrainStem** | Max | ≤54Gy |
| **BrainStem_PRV** | D1^‡^ | ≤60Gy |
| **OpticNerves_L** | Max | ≤50Gy |
| **OpticNerves_L PRV** | D1^‡^ | ≤54Gy |
| **OpticNerves_R** | Max | ≤50Gy |
| **OpticNerves_R PRV** | D1^‡^ | ≤54Gy |
| **Chiasm** | Max | ≤50Gy |
|  | D1^‡^ | ≤54Gy |
| **Lens_L** | Max | <25Gy |
| **Lens_R** | Max | <25Gy |
| **Parotid _L** | Mean | < 26 Gy |
|  | V30^§^ | <50% |
|  | V20* | >20cc |
| **Parotid _R** | Mean | < 26 Gy |
|  | V30& | <50% |
|  | V20^*^ | >20cc |
| **TemporalLobe_L** | Max | ≤60Gy |
| **TemporalLobe_R** | Max | ≤60Gy |
| **Mandible_L** | D1cc^&^ | <75Gy |
| **Mandible_R** | D1cc^&^ | <75Gy |
| **TM Joint_L** | D1cc^&^ | <75Gy |
| **TM joint_R** | D1cc^&^ | <75Gy |
| **Larynx** | Mean | <45 Gy |
| **Cochlea_L** | Mean | <50Gy |
| **Cochlea_R** | Mean | <50Gy |

¶: Percentage dose covering 93% of the PTV

#: Percentage dose covering 100% of the PTV

†: Percentage volume that received > 110% of the Rx (prescribed dose)

||: Percentage volume that received > 115% of the Rx (prescribed dose)

‡: The dose received by 1% of the volume.

§: The percentage volume of at least one gland which received >30 Gy.

*: The volume of both glands which received < 20 Gy.

&: Dose received by 1cubic centimeter of the volume.
